# Food Protein-Induced Allergic Proctocolitis: Real-World Experience from an Italian Cohort

**DOI:** 10.3390/nu17010098

**Published:** 2024-12-30

**Authors:** Simona Barni, Benedetta Pessina, Lorenzo Fioretti, Luca Scarallo, Andrea Di Siena, Matteo Bramuzzo, Giulia Liccioli, Lucrezia Sarti, Leonardo Tomei, Mattia Giovannini, Sara Renzo, Francesca Mori

**Affiliations:** 1 Allergy Unit, Meyer Children’s Hospital IRCCS, 50139 Florence, Italy; 2 Department of Health Sciences, University of Florence, 50139 Florence, Italy; 3 Gastroenterology and Nutrition Unit, Meyer Children’s Hospital IRCCS, 50139 Florence, Italy; 4 Department of NEUROFARBA, University of Florence, 50139 Florence, Italy; 5 Division of Pediatrics, Department of Medicine (DAME), University of Udine, 33100 Udine, Italy; 6 Pediatric Gastroenterology, Digestive Endoscopy and Clinical Nutrition Unit, Department of Pediatrics, Institute for Maternal and Child Health IRCCS “Burlo Garofolo”, 34137 Trieste, Italy

**Keywords:** non-IgE-mediated food allergy, food protein-induced allergic proctocolitis, proctocolitis, culprit food, hematochezia, infant, elimination diet, cow’s milk, hen’s egg, bloody stool

## Abstract

**Background/Objectives**: Food protein-induced allergic proctocolitis (FPIAP) is a non-IgE-mediated food allergy, usually presenting as bloody stools in breastfed, well-appearing, and regularly growing infants. The aim of our study was to describe the clinical features of Italian infants affected by FPIAP and their management and natural history in a real-life setting. **Methods**: A retrospective, observational study was performed at two tertiary pediatric hospitals (Florence and Trieste), including FPIAP-diagnosed infants between 2012 and 2022. **Results**: Most of the 100 enrolled patients were breastfed (68.0%), and the majority of those who underwent diagnostic tests (*n* = 51) showed normal hemoglobin and total IgE levels. A maternal elimination diet was performed in 69.0%, mostly for milk only, but 40.6% underwent multiple elimination diets. The remission rate was high both in breastfed infants (76.8%) and in those who received extensively hydrolyzed formula (81.8%). Nine subjects were left on a free diet, but six were lost at follow-up. The median time of complete remission was 30 days (IQR 14–60). Culprit food reintroduction was tolerated at a median age of 8 months (IQR 6–11), in ladder modality (for hen’s egg and cow’s milk) in 61.7%. Nine patients relapsed (14.3%) upon reintroduction with no associated variables identified at the regression analysis. The relapse rate was slightly higher when trigger food reintroduction was attempted > 12 months (16.7%) versus <12 months (13.0%). **Conclusions**: In our population, FPIAP had, as expected, a benign evolution. The early reintroduction of the suspect food in a gradual manner for cow’s milk and hen’s egg leads to good tolerance within the first year in most patients, avoiding unnecessary elimination diets.

## 1. Introduction

Food protein-induced allergic proctocolitis (FPIAP) is a non-IgE-mediated food allergy, presenting as bloody stools in healthy, well-growing infants [1,2,3]. Diagnosis relies on clinical manifestations, resolution with the elimination of the trigger food, and symptom recurrence upon its reintroduction into the diet [1].

The primary culprit food in breastfed infants is cow’s milk (CM), though other triggers, such as hen’s egg (HE) (22.2–37.4%), beef (8–11%), wheat (5.5%), chicken meat (2.4%), fish (2.4%), and treenuts (1.2–3.3%), have been described [4,5,6,7,8,9,10]. Other less common triggers are lamb or turkey, legumes, and rice [5,10], while, in formula-fed infants, the most frequent triggers are CM and soy [11]. Multiple food allergy (multiple FPIAP) occurs in 35% to 42.9% of cases [5,10] and has been linked to peripheral eosinophilia and atopic dermatitis [5,9], complicating management also due to the possible employment of extensive elimination diets. Laboratory tests are usually normal in FPIAP patients, even though higher eosinophil levels have been reported in severe cases [10].

Even though clinical studies, position papers, and guidelines have been recently published by several scientific societies, the usefulness of an elimination diet is still debated [1,12,13,14,15,16], diet protocols are highly variable [17], and diet duration and the modality of food reintroduction are not uniform in clinical practice [18].

The prognosis of this condition is usually good. Although FPIAP generally resolves before age one, late tolerance (>12 months) is observed in roughly 30% of patients [10] and may be associated with late complementary feeding (e.g., weaning after 5.5 months) and atopic background (e.g., atopic dermatitis) [10]. Despite the growing literature on this topic, a lack of standardization in the diagnosis and management of these patients has been recently brought to light by survey studies [19], and the need for action has emerged [20].

The aim of our study was to describe the demographic and clinical features of infants diagnosed with FPIAP followed-up by two different Italian pediatric tertiary centers, providing an overview on nutritional management strategies, prognosis, and natural history in our population.

## 2. Materials and Methods

### 2.1. Study Design and Population

This retrospective, observational study included patients followed-up by two Italian pediatric hospitals (Meyer Children’s Hospital IRCCS, Florence, and Burlo Garofolo Hospital IRCCS, Trieste) and diagnosed with FPIAP between January 2012 and December 2022. Patients were screened from the hospital databases by two separate physicians, using the following search strings: “melena and rectal bleeding”, “allergic colitis and gastroenteritis”, and “non-infectious colitis”. We only selected patients with FPIAP diagnosis confirmed with the following inclusion criteria: suggestive symptoms and exclusion of other causes of rectal bleeding (e.g., nipple fissures), the temporary resolution of hematochezia upon the elimination of a suspected food, the recurrence of hematochezia upon the oral food challenge performed with the same suspect food [1]. The exclusion criteria were failure to provide informed consent, confirmed alternative cause of rectal bleeding, and absent information in the medical record. The code of the event report issued by Meyer Children’s Hospital IRCCS is IR904-24-72299, and written informed consent was obtained from the parents of participating patients.

### 2.2. Data Collection from Medical Records

We extracted information about gender, age, clinical symptoms, family/personal history of allergic comorbidities, history of preterm birth or perinatal comorbidity, and type of lactation (breastfeeding, artificial, or mixed lactation) from medical records. Information about laboratory tests (blood tests with complete blood count and hemoglobin levels, stool tests including screening for *Adenovirus*, *Rotavirus*, and culture), colonoscopy, and allergy tests (total and specific IgE testing and/or skin prick tests—SPTs) was also collected when these were performed. IgE-mediated sensitization was defined when either SPTs for food allergens, using the technique described by Heinzerling et al. [21], showed a wheal with diameter ≥ 3 mm compared to negative control or when the specific IgE level was ≥0.35 kU/L. Total IgE and specific IgE levels were determined using a commercial assay (ImmunoCAP system, Thermo Fisher Scientific, Waltham, MA, USA). Eosinophilia was defined when the peripheral eosinophil count was ≥500/mm^3^. “Severe FPIAP” was defined according to the existing literature when patients showed either abundant bleeding (mild-moderate anemia), severe perianal disease, abdominal distension, growth failure, or poor general conditions [10,12].

Data on the therapeutic approach adopted in the patients enrolled were retrieved. Three possible management approaches were adopted: (1) maternal trigger food elimination diet, (2) extensively hydrolyzed/aminoacidic formula, or (3) no elimination diet. “Remission” was defined as the complete disappearance of all symptoms. In some cases of maternal elimination diet, the elimination of multiple trigger foods was indicated, because no remission occurred with a single-food elimination diet. The order of eliminated foods was, according to previously published protocols [22], the frequency of food sources in the Italian diet and the probability of triggering FPIAP, first CM, then HE/soy, then wheat/fish. Each food was subsequently eliminated waiting at least 72–96 h if no clinical improvement was seen (e.g., persisting or worsening symptoms), otherwise waiting 7–14 days before removing the next suspected food if clinical improvement was partial (e.g., reduction in the number of bloody evacuations or in the amount of blood at each evacuation).

The elimination diet lasted until the child was 10–12 months of age or less in case the child accidentally consumed the trigger food and had no recurrence of symptoms; then, culprit food reintroduction was attempted into the child’s diet. For CM and HE, reintroduction occurred from baked to heated to fresh food (“ladder” modality) [23]; otherwise, the fresh food was directly given to the child (e.g., soy). Reintroduction could take place at home or in the hospital setting upon the request of parents scared to reintroduce the culprit food at home. When multiple foods were eliminated, reintroduction started from the last eliminated food and backwards, until the patient showed any further clinical symptoms or until all the eliminated foods were tolerated. In case of symptom recurrence upon reintroduction, patients were instructed to continue the elimination diet for 3 months before a second attempt, similar to previously published studies [24]. If the child showed a recurrence of symptoms at the reintroduction attempt, we defined this as “relapse”. If the child did not show a recurrence of symptoms at the reintroduction attempt, we defined this as “tolerance” and considered FPIAP resolved. “Full tolerance” was defined when the child was able to consume all forms of the trigger food without relapse, and it was defined early when occurring <12 months and late when occurring ≥12 months.

### 2.3. Data Collection from Telephonic Survey

The patients’ parents were telephonically surveyed about the timing and modality of the reintroduction of the culprit food, the success of the reintroduction attempt, and the future development of any atopic comorbidity (asthma, oculorhinitis, atopic dermatitis, IgE-mediated food allergy).

### 2.4. Statistical Analysis

Statistical analyses were performed using IBM Statistical Package for Social Science software (SPSS, Version 28.0, Chicago, IL, USA). Non-normally distributed variables were presented as medians (interquartile ranges, IQRs), while categorical variables were presented as percentages. We performed the unpaired Wilcoxon–Mann–Whitney test to assess differences among quantitative continuous variables. The analyses of statistical differences among categorical variables were carried out using the Chi-square test. To identify clinical predictors for relapse, we performed a binary logistic regression analysis. The relapse of hematochezia upon the reintroduction of the culprit food was selected as the outcome (dependent) variable. Univariate analyses identified predictors (independent variables) for the multivariable model, fitted using a backward elimination procedure (likelihood ratio was used as criteria and *p* > 0.05 for removal). The Hosmer–Lemeshow test was used to evaluate the goodness of fit of the multivariable model. All statistical tests were two-sided, and *p* < 0.05 was considered as the statistically significant threshold.

## 3. Results

### 3.1. Patients’ Selection

Of 3069 medical charts screened, a total of 100 cases fulfilled the diagnostic criteria for FPIAP and were included, 10 from Burlo Garofolo Hospital IRCCS, Trieste, and 90 from Meyer Children’s Hospital IRCCS, Florence.

### 3.2. Demographic and Clinical Characteristics at Symptom Onset

The main demographic and clinical characteristics of our cohort are shown in Table 1. The median age of symptom onset was 2 months, with equal gender distribution. Atopic dermatitis and a family history of atopy were present in 10.0% and 20.0% of cases, respectively. Almost all patients showed bloody stools as the main presenting symptom (99.0%), associated with mucus in the stools in half of the patients. Less common symptoms were diarrhea, abdominal gas, vomiting, and abdominal pain. At the physical examination, an anal fissure was associated in 22.0% of cases. Only a minority of the patients (6.0%) showed failure to thrive.

### 3.3. Laboratory and Diagnostic Work-Up Procedures

Diagnostic exams were performed in 51.0% of patients (Table 2). Most infants showed normal hemoglobin (Hb) levels, with a median value of 11.0 g/dL (IQR 10.3–12.5 g/dL). A slightly elevated eosinophil count was observed, with a median value of 543 eosinophils/mm^3^ (IQR 340.0–1695.0) and normal median total IgE levels (5.0 kU/L, IQR 2.0–19.0). Median eosinophil level was not different in the severe versus mild FPIAP cases (*p* = 0.650). No patients showed IgE-mediated sensitization, and all infectious etiological investigations were negative. Eight patients (8.0%) underwent a surgical specialistic evaluation to rule out other causes of rectal bleeding, and another 8.0% underwent colonoscopy. All the patients who underwent colonoscopy either showed peripheral eosinophilia, abdominal symptoms different from bloody or mucous stool, or a late response to the elimination diet. The endoscopic and histological findings were not available for six patients, because they had been examined in other hospitals, and the report was not given to their families; in the only two available reports, recto-sigmoid nodular lymphoid hyperplasia associated with eosinophils infiltration of the lamina propria at biopsy was present.

### 3.4. Therapeutic Approach and Response to Elimination Diet

Most patients were exclusively breastfed (68.0%), with 23.0% on mixed lactation and 9.0% receiving exclusively formulated milk. Different therapeutic strategies were adopted in our cohort:

1. Maternal elimination diet (69.0%): of these patients, 52 were exclusively breastfed and 17 were on mixed lactation.
2. Extensively hydrolyzed formula (eHF) (22.0%): of these, 7 were exclusively breasted, 6 were on mixed lactation, and 9 were on exclusive formula.
3. No dietary changes (9.0%): of these, all were exclusively breastfed.

Regarding maternal diet, the most frequently eliminated food was CM and its derivatives. Table 3 shows the elimination diet trends adopted in our cohort. The most common approach was a single-food elimination diet, while a multiple elimination diet was performed in 40.6% of cases (*n* = 28/69), mainly eliminating CM and HE or soy. Cases of three-, four-, or five-food elimination diets were rarer. The median eosinophil count in the subgroup of children with multiple FPIAP was the same as single-food FPIAP patients (*p* = 0.628), and only one had atopic dermatitis.

When assessing the response to maternal diet, remission was achieved in 76.8% of the patients (*n* = 53/69), while in 17.4% (*n* = 12/69), it was not possible to evaluate the response to the diet, because patients were lost to follow-up. In only 5.8% (*n* = 4/69), remission was not obtained with the maternal diet, and artificial lactation with an extensively hydrolyzed formula was indicated, with a resolution of symptoms in all cases. The median time of complete symptom remission in the case of the maternal diet was 30.0 days (IQR 14.0–60.0), significantly higher in patients with multiple FPIAP (median 47.5 days, IQR 30.0–90.0, *p* = 0.045). Median time to remission increased progressively from patients eliminating CM and soy (median time of symptoms disappearance 30.0 days), wheat (37.5 days), HE (47.5 days), and finally, fish (60.0 days), which partially reflects the timing of progressive food elimination performed in our cohort. When the first therapeutic measure adopted was the use of eHF, the rate of remission was very high (81.8%, *n* = 18/22), while, in non-responders to eHF, an aminoacidic formula was employed with the complete resolution of symptoms (18.2%, *n* = 4/22). The median time of complete remission when using the hydrolyzed formula was 15.0 days (IQR 1.0–30.0).

### 3.5. Follow-Up and Outcomes

Sixty-one patients underwent a telephonic follow-up survey. Thirty-nine patients were lost to follow-up. When the elimination diet was performed, the median duration was 5.3 months (IQR 2.5–7.0). All patients underwent a normal weaning, at a median age of 6 months (IQR 5.25–6.00). Unfortunately, of the nine infants who did not receive treatment, incomplete information was retrieved from clinical charts; three were normally weaned at 6 months; and only one of them showed protracted bleeding, which stopped at 23 months. The other six patients were lost to follow-up. 

Overall, trigger food reintroduction was achieved in most patients (98.4%, *n* = 60/61). In one case (1.6%), the culprit food (HE) reintroduction caused multiple relapses, and at 1 year and 8 months of age, the patient developed acute food protein-induced enterocolitis syndrome (FPIES). Trigger food reintroduction was mainly performed at home (95.0%, *n* = 57/60) and only in a few cases in the hospital setting (5.0%, *n* = 3/60). At reintroduction, SPTs were performed in only eight patients (12.7%), and the results were persistently negative.

The median age of the first reintroduction attempt was 8.0 months (IQR 6.0–11.0). In most patients (76.7%, *n* = 46), it occurred < 12 months of age, being unsuccessful (patients showed a relapse of typical symptoms) in 13.0% (*n* = 6/46). In a few patients, the first reintroduction attempt was delayed ≥ 12 months (26.2%, *n* = 12), and it was unsuccessful in 16.7% (*n* = 2/12). In two patients, the timing of the reintroduction was unknown. Therefore, eight patients relapsed at the first attempt of trigger food reintroduction (13.3%), but no variable was predictive of relapse in the logistic regression analysis (Table 4).

In 37 patients (61.7%), reintroduction was performed in the ladder modality; whereas, another 19 patients (31.7%) reintroduced the fresh food directly. In four patients, the modality of reintroduction was unknown. No statistically significant difference in relapse rate was observed according to reintroduction timing or modality. The full tolerance of the trigger food was achieved in 43 patients before reaching the first year of life (71.7%), in 15 patients within the second year (25.0%), and in two patients after the second year (3.3%). We observed no differences between early and late tolerant patients with regards to clinical or demographical characteristics (Table 5). The latest age for full tolerance in our cohort was 35 months.

**Figure 1:**
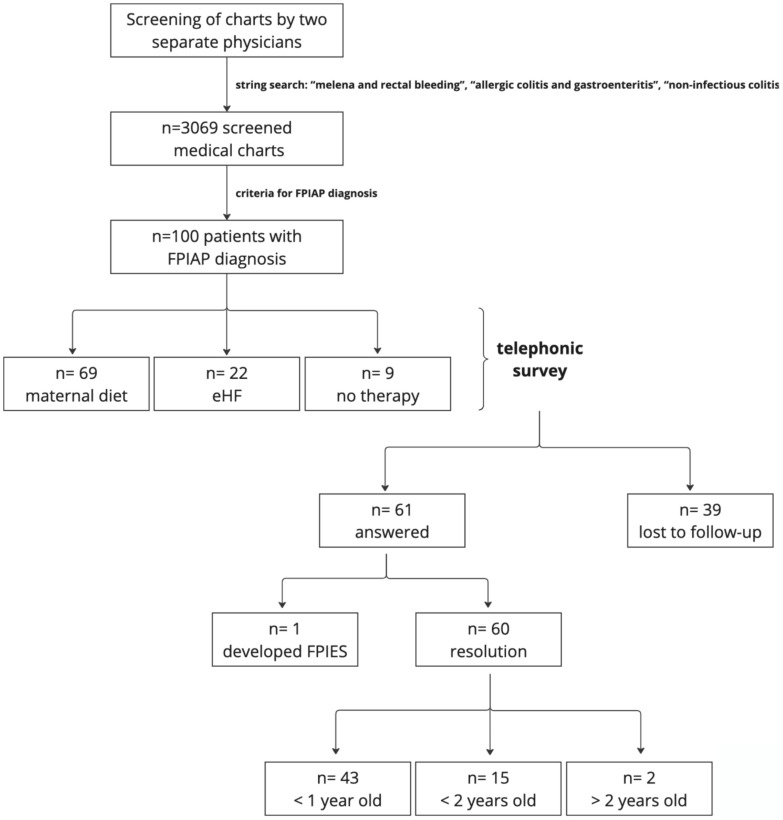
Diagram illustrating the study design and results.

During follow-up, only one patient (1.6%) developed an atopic comorbidity (house dust mite sensitization and recurrent wheezing), but none showed a conversion to IgE-mediated food allergy. Figure 1 summarizes the study design and its results.

## 4. Discussion

This is the first multicenter study investigating the real-world characteristics and management of FPIAP patients in the Italian population. It aimed to describe the demographic–clinical features and the diagnostic–therapeutic approaches, prognosis, and natural history of Italian children affected by FPIAP, which are missing data in the literature so far.

We described an early clinical presentation (around 2 months of age), mainly in breastfed infants and attributed to CM as primary trigger, followed by HE, soy, fish, and wheat, consistent with Italian dietary habits and prior studies [4,5,6,7,8,9,25,26]. About 40% of patients had multiple trigger food allergens, aligning with previous reports (5 to 42%) [5,6,7,8,9,10,27]. Unlike Turkish studies [5,9], our cohort showed no link between multiple FPIAP and concomitant atopic dermatitis or high eosinophils levels at diagnosis. However, atopic comorbidity was generally low in our population, with only 10% affected by atopic dermatitis, versus 22–52% in the literature [25,26,28], and 20% with atopic family history, lower than previously reported (25–50%) [25,26,28]. While median eosinophil counts were slightly elevated in our cohort, this was not associated with severe FPIAP, which is typically also associated with earlier clinical manifestation onset, anemia, and neutropenia [10]. Therefore, the higher median eosinophil count was not a marker of severity in our population, but better designed studies are needed to establish this; moreover, studies comparing eosinophil count in FPIAP patients and healthy controls or infants with hematochezia, but not FPIAP, could be useful to understand if this is a marker of disease at all.

As previously observed [1,2,3,5,9,26], the most common presenting symptom was bloody stools in a regularly growing infant, while diarrhea, abdominal gas, vomiting, and abdominal pain were less frequent. Bloody stools without mucus was present in half of our population, similar to another study [25], possibly due to the higher noticeability of hematochezia for parents when compared to mucous stools. Few patients exhibited failure to thrive, and most of these underwent diagnostic investigations. Interestingly, despite a recent survey conducted by the Italian Society of Pediatric Gastroenterology and Nutrition showing that most pediatricians do not recommend in-depth examinations when FPIAP is suspected [19], half of our patients underwent at least one diagnostic exam. This observation highlights a gap between survey studies and real-world patient management. Nonetheless, the median hemoglobin level was normal in our cohort, even though anemia has been previously described in patients with persistent bleeding [25]. In our patients who underwent allergic investigations, no IgE sensitization was found. The possibility of an increase in the total IgE levels in FPIAP has been previously reported [25,26,29,30], and allergic sensitization to trigger foods in FPIAP infants ranges from 10 to 35% [9,26], known to be a risk factor for multiple FPIAP [9] and for late resolution [27]. Due to the lack of IgE sensitization in our cohort, we could not explore these aspects in our cohort. A minority of the patients underwent a surgical evaluation or endoscopy to rule out other causes of rectal bleeding. According to existing evidence, endoscopy is not recommended in patients with FPIAP, except for those who do not respond to the maternal elimination diet or to aminoacidic formula or for those with severe symptoms. In our cohort, all patients undergoing colonoscopy were either refractory to diet or had some features of severe FPIAP.

The need of an elimination diet is still debated, and only one randomized controlled trial addressed this gap in the knowledge [11], while for now, the decision relies on the clinical experience of physicians and the will of their patients. The median time for symptom remission following a maternal diet was 30.0 days, longer for patients with multiple FPIAP. However, this observed increase is biased by the elimination diet indications given by physicians, which includes a 3–14 days interval of observation before the removal of a further suspect trigger. Due to the retrospective nature of the study, the precise timing of symptom resolution after the final food elimination could not be determined, and this is one limitation of our study. Nonetheless, complete remission rate was high, both when maternal diet was adopted and when eHF was used; 18% of patients required switching to an amino acid formula, slightly higher than the 10% reported in the literature [26,28,31], likely reflecting our tertiary care cohort, which may include more severe cases. For the same reason, only nine patients did not undergo any diet modification, and unfortunately, six of them were lost to follow-up.

Mothers were on an elimination diet for an average of 5 months. Trigger food reintroduction was attempted at a median age of 8 months, and in 13.3% of patients, it was unsuccessful at the first attempt. The age of onset, comorbidity, prematurity, and family history of atopy were not associated with a relapse of symptoms. Some authors [14,30] suggest performing SPT to the trigger food to check the negativity before the reintroduction at home. In our study, trigger food reintroduction was successful in most patients (98.4%), occurring mainly at home (95.0%) and with negative SPTs, which were rarely performed at all.

Not only is the necessity of an elimination diet still debated, but also the time and modality of the offending food reintroduction are not uniformly codified and vary considerably according to the studies [1,12,13,14,15,16]. Most patients in our cohort underwent gradual reintroduction following the food ladder for CM and HE, as suggested by Venter et al. [32]. The relapse rate was not significantly different according to modality or timing of reintroduction; however, a trend seems to exist, with a slightly higher relapse rate with a later attempt compared to an earlier attempt (16.7% ≥12 months versus 13.0% <12 months). We may speculate that an early allergen reintroduction could be tolerated because of immune system immature priming and the possibly higher tolerogenic potential in younger infants, as already hypothesized for IgE-mediated allergy [33]. In our population, all patients underwent a normal weaning at a median age of 6 months, while 45% of pediatricians tend to change the order in which foods are introduced into an infant’s diet during weaning, causing a delay in the introduction of certain types of foods with the risk of developing IgE-mediated food allergies [19].

Full tolerance to the trigger food was achieved in 71.7% of patients within the first year, 25.0% by the second year, and 3.3% after the second year of life in line with the study conducted by Kaya et al. that reported tolerance acquisition in 53.0% at 1.25 years, 25.0% at 2 years, and finally, 1.7% at 3 years [7]. No significant differences were observed in the early versus late tolerant individuals, and none of our patients showed a conversion to IgE-mediated food allergy in contrast with two studies that found that 3.6% and 11.0% of patients developed an IgE-mediated allergy to the trigger food over time, respectively [34,35]. However, one of our patients developed FPIES to HE (the same trigger food for FPIAP).

Our study has some inherent limitations. It is a retrospective study spanning 10 years, involving multiple centers and physicians, which may introduce data inconsistencies. Selection bias is also possible, as tertiary centers are more likely to see severe cases and may exclude milder cases in which the elimination diet and investigations are not usually performed. Additionally, we described a relatively short follow-up period with a high percentage of patients lost to follow-up (39.0%). These limitations reflect the “real-world” scenario, which differs from single-center, prospective studies. Despite this, we feel that this population is representative of FPIAP management in Italy over the past 10 years, providing interesting real-life data to the limited literature of Italian infants affected by FPIAP. Our study largely confirmed the information already present in the literature thus far, adding more data about trigger food reintroduction modality and timing, which are often lacking in other studies. This information should serve as a hint to perform further studies with the aim of defining a validated guideline for clinicians dealing with FPIAP patients, in order to avoid unnecessary investigations and protracted elimination diets. On the contrary, our data did not confirm the association of multiple food triggers with atopy or eosinophils levels, which were overall elevated in our cohort. These controversies should be solved by more prospective studies searching for markers of severity of disease in order to individualize the FPIAP management strategy.

## 5. Conclusions

FPIAP is a benign condition predominantly affecting well-growing breastfed infants. Single-food FPIAP (particularly CM) is more frequent than multiple FPIAP. The elimination of the trigger food from the mother’s diet or switching to eHF in formula-fed infants leads to symptom remission in most cases. Prognosis is favorable, with most patients tolerating trigger foods within the first year. The early reintroduction of the trigger food and a normal weaning appear reasonable for non-severe cases. Further prospective studies are necessary to management protocols, particularly regarding the indication to maternal diets and the modality and timing of reintroduction of the culprit food.

## Figures and Tables

**Table 1:** Demographic and clinical characteristics of our cohort. IQR: interquartile range; NICU: neonatal intensive care unit; mo: months; *n*: number.

Characteristic		*n* (Total *n* = 100)
Female gender, %	50.0	50
Age of onset (mo), median (IQR)	2.0 (1.0–3.0)	100
Age of diagnosis (mo), median (IQR)	3.53 (2.0–5.0)	100
Bloody stool, %	99.0	99
Mucus in stool, %	50.0	50
Diarrhea, %	20.0	20
Vomiting, %	4.0	4
Abdominal pain, %	4.0	4
Anal fissure, %	22.0	22
Normal growth, %	94.0	94
Atopic dermatitis, %	10.0	10
Preterm birth, %	5.0	5
NICU hospitalization, %	13.0	13
Family history of atopy, %	20.0	20

**Table 2:** Laboratory investigation frequencies in our cohort. *n*: number.

Investigations	%	*n* (Total *n* = 100)
Any	51.0	51
Complete blood count	44.0	44
Fecal tests	30.0	30
Skin prick tests	25.0	25
Total and specific IgE	21.0	21
Colonoscopy	8.0	8
Surgical consult	8.0	8

**Table 3:** Diet elimination trends in our cohort. *n*: number.

Offending Food	%	*n* (Total *n* = 69)
Cow’s milk (CM)	98.5	68
Hen’s egg (HE)	34.8	24
Soy	13.0	9
Fish	5.8	4
Wheat	4.3	3
**Single food**	59.4	41
CM		40
HE		1
**Two foods**	30.4	21
CM and HE		16
CM and soy		5
**Three foods**	5.8	4
CM, HE, and soy		2
CM, HE, and wheat		1
CM, HE, and fish		1
**Four foods**	2.9	2
CM, HE, fish, and wheat		1
CM, HE, soy, and fish		1
**Five foods**	1.4	1

**Table 4:** Logistic regression analysis for variables associated with relapse. CI: confidence interval; FPIAP: food protein-induced allergic proctocolitis; NICU: neonatal intensive care unit; OR: odds ratio; *p*: *p* value considered significant when *p* < 0.05.

Characteristic	Unadjusted OR (95% CI)	*p*-Value	Adjusted OR (95% CI)	*p*-Value
Age of onset (months)	0.755 (0.410–1.392)	0.368	0.817 (0.397–1.683)	0.584
Gender (male vs. female)	0.521 (0.117–2.322)	0.392		
Family history of atopy (yes vs. no)	1.400 (0.259–7.582)	0.696		
Atopic dermatitis (yes vs. no)	0.488 (0.082–2.921)	0.432		
NICU hospitalization (yes vs. no)	0.488 (0.082–2.921)	0.432		
Multiple FPIAP (yes vs. no)	1.859 (0.347–9.956)	0.469		
Reintroduction modality (gradual vs. free)	1.133 (0.188–6.847)	0.892		
Age at first reintroduction attempt (months)	1.045 (0.840–1.301)	0.692		
Age at weaning (months)	0.571 (0.163–1.996)	0.380	0.546 (0.156–1.905)	0.342

**Table 5:** Differences between patients with early or late tolerance. FPIAP: food protein-induced allergic proctocolitis; mo: months; n: number; SD: standard deviation.

Characteristic	Patients with Early Tolerance (*n* = 43)	Patients with Late Tolerance (*n* = 15)	*p*-Value
Gender			0.564
Male (%) Female (%)	19 (44.2) 24 (55.8)	8 (53.3) 7 (46.7)	
Mean age of onset, months (SD)	1.9 (1.2)	2.2 (1.5)	0.435
Family history of atopy	14 (32.6)	1 (6.7)	0.084
Atopic dermatitis	4 (9.3)	4 (26.7)	0.185
Multiple FPIAP	15 (34.9)	3 (20.0)	0.348
Mean eosinophil count, cells/mmc (SD)	915.0 (989.6)	913.4 (742.9)	0.997

## Data Availability

The data presented in this study are available on request from the corresponding author to guarantee the privacy of the participants, whose research data are confidential.
